# General information for patients and carers considering haematopoietic stem cell transplantation (HSCT) for severe autoimmune diseases (ADs): A position statement from the EBMT Autoimmune Diseases Working Party (ADWP), the EBMT Nurses Group, the EBMT Patient, Family and Donor Committee and the Joint Accreditation Committee of ISCT and EBMT (JACIE)

**DOI:** 10.1038/s41409-019-0430-7

**Published:** 2019-01-31

**Authors:** Helen Jessop, Dominique Farge, Riccardo Saccardi, Tobias Alexander, Montserrat Rovira, Basil Sharrack, Raffaella Greco, Nico Wulffraat, John Moore, Majid Kazmi, Manuela Badoglio, Gillian Adams, Bregje Verhoeven, John Murray, John A. Snowden

**Affiliations:** 1 Department of Haematology, Sheffield Teaching Hospitals NHS Foundation Trust, Sheffield S10 2JF, UK; 2 Unité de Médecine Interne: Maladies Auto-immunes et Pathologie Vasculaire (UF 04), Hôpital St-Louis, AP-HP, 1 avenue Claude Vellefaux, Paris, France; 3 Centre de Référence des Maladies auto-immunes systémiques Rares d’Ile-de-France, Filière FAI2R, Paris, France; 4 EA 3518, Université Denis Diderot, Paris 7, France; 5 Department of Internal Medicine, McGill University, Montréal, Canada; 6 Haematology Department, Careggi University Hospital, Florence, Italy; 7 Klinik fur Rheumatologie und Klinische Immunologie, Charite-Universitatsmedizin, Berlin, Germany; 8 Department of Haematology, Institute of Haematology and Oncology, IDIBAPS, Hospital Clinic, University of Barcelona, Josep Carreras Leukaemia Research Foundation, Barcelona, Spain; 9 Department of Neurology, Sheffield Neuroscience BRC, Sheffield Teaching Hospitals NHS Foundation Trust, University of Sheffield, Sheffield S10 2JF, UK; 10 Hematology and BMT Unit, San Raffaele Scientific Institute, Via Olgettina 60, 20132 Milano, Italy; 11 Divisie Kinderen, Cluster Immunologie, Reumatologie, Hematologie en Infectiologie, Wilhelmina Kinderziekenhuis, Utrecht, The Netherlands; 12 Department of Haematology, St Vincents Hospital Sydney, Darlinghurst, NSW Australia; 13 Kings Healthcare Partners, Department of Haematology, Guys Hospital, London SE1 9RT, UK; 14 EBMT Paris study office/CEREST-TC, Department of Haematology, Saint Antoine Hospital – INSERM, Paris, France; 15 EBMT Executive Office, Eddific Dr. Frederic Duran i Jorda, Passeig Taulat, 116. 08005, Barcelona, Spain; 16 Christie Hospital NHS Foundation Trust, Manchester, UK

**Keywords:** Stem cells, Autoimmune diseases

## Abstract

Over the last 20 years, haematopoietic stem cell transplantation (HSCT) has been used to treat patients with severe autoimmune and inflammatory diseases whose response to standard treatment options has been limited, resulting in a poor long-term prognosis in terms of survival or disability. The vast majority of patients have received autologous HSCT where an increasing evidence-base supports its use in a wide range of autoimmune diseases, particularly relapsing remitting MS, systemic sclerosis and Crohn’s disease. Compared with standard treatments for autoimmune diseases, HSCT is associated with greater short-term risks, including a risk of treatment-related mortality and long-term complications. There is a need for a careful appraisal of potential benefits and risks by disease and transplant specialists working closely together with patients and carers to determine individual suitability for HSCT. HSCT should be conducted in accredited transplant centres with robust arrangements for long-term follow-up with both disease and transplant specialists. The aim of this open-access position statement is to provide plainly worded guidance for patients and non-specialist clinicians considering HSCT for an autoimmune disease, especially when treatment abroad is being considered. Recent technical publications in the field have been referenced to support the statement and provide more detail for clinicians advising patients.

## Introduction

Autoimmune diseases (ADs) are a broad group of illnesses where the body’s immune system reacts against its own tissues and organs. The immune attack is followed by chronic inflammation and abnormal healing, which may be associated with permanent organ damage, disability and poor quality of life. In some cases, severe ADs may shorten life expectancy or even be immediately life-threatening.

The types of organs affected vary between ADs. For example, systemic sclerosis, lupus, vasculitis and other connective tissue diseases affect many organs, typically causing inflammation and scarring of the skin, heart, lungs, kidneys and other organs. Multiple sclerosis (MS) affects the brain and spinal cord, whereas Crohn’s disease affects the gut.

Treatment with immunosuppressant drugs, including disease modifying treatments (DMTs), may be successful in controlling the AD, but there is an increased susceptibility to infection and organ damage, which add to the problems of living with an AD.

Some patients have very aggressive forms of autoimmune disease which are poorly controlled by standard therapies. In some of these severely affected patients, there may be benefit in considering bone marrow transplantation (BMT), or, as it is now more commonly known, haematopoietic stem cell transplantation (HSCT) [1–3].

## HSCT: general background

HSCT is commonly used for the treatment of serious blood diseases, including leukaemia, myeloma and lymphomas. Many haematology departments will provide HSCT as a treatment for these common indications, having carefully weighed up the survival benefits and serious risks of HSCT against the non-transplant treatment options, such as chemotherapy and modern biological agents [4, 5].

The process of HSCT involves giving high doses of chemotherapy and/or radiotherapy and then infusing mobilized blood, cord blood or marrow-derived (i.e.,’ haematopoietic’) stem cells from the patients themselves (autologous or auto-transplant) or from donors (allogeneic or allo-transplant) to rebuild the bone marrow and the immune system, hopefully without the disease.

**Fig. 1:**
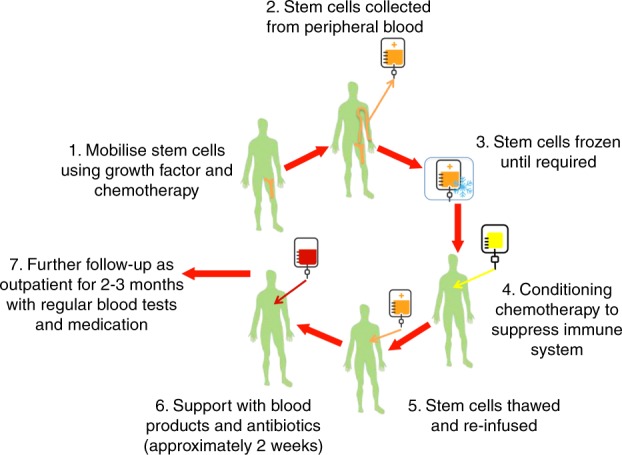
Summary of autologous HSCT procedure. In addition, robust long-term follow-up arrangements should be individualised with each patient with both disease and transplant specialists

The HSCT process is summarised in Fig. 1. Recognised early complications include acute toxicities experienced during the procedure, such as infection, bleeding, anaemia and organ toxicities. Blood transfusions are usually needed to support the patients during this phase of the treatment. Complications may be life-threatening and irreversible and therefore HSCT should only be performed after a full discussion of the risks and how they may be justified in terms of potential benefits from the treatment. Treatment-related mortality (TRM) risks depend on the type of transplant, the disease being treated and the individual patient. Even in patients treated in accredited transplant centres, TRM risk ranges from around 1% in good risk patients undergoing autologous HSCT to considerably higher in patients with more risk factors (e.g., severe organ damage or disability) and with allogeneic HSCT, where TRM risk commonly exceeds 10%. Responsible treating clinicians need to openly discuss and individualise these risks for patients as part of the informed consent process.

The risks of the HSCT procedure are not limited to the inpatient phase prior to recovery of blood counts [6–8]. Following hospital discharge after the HSCT procedure, given the risk of ongoing complications, especially the risk of sudden onset of serious and life-threatening complications including infection, there should always be robust plans for follow up and urgent management of complications. Patients should be able to self-refer into a haematology or other department familiar with HSCT on a 7-day, 24-h basis, and the transplant unit should organise regular outpatient monitoring in the first 2 years following the procedure and thereafter if needed. The risks of infection persist for long periods after HSCT, and ‘prophylactic’ (preventative) antibiotics are recommended for many months, or sometimes even life-long. Even though patients may feel well, some complications, including certain types of pneumonia, may be rapid in their onset and progression and much more challenging to treat when established. Patients need to be monitored, and provided with a consistent supply of prophylactic (preventative) antibiotics, which reduce hospitalisation for serious and sometimes life-threatening complications. Vaccination should be considered according to current guidelines for immunocompromised patients [9].

Long-term monitoring is essential. The so-called ‘late effects’ of HSCT are common and may involve many organ systems, including glandular (endocrine), bone, cardiovascular, respiratory and neurological. HSCT can also result in an increased risk of ‘new’ cancers, and have an impact on patients’ psychological and emotional well-being. Reproductive impairment can be frequent, and programmes for prevention of chemotherapy-associated sterility should therefore be proposed prior to HSCT. Allogeneic HSCT may also be associated with risks of graft-versus-host disease, a potentially severe immune complication that may affect several organs. There is even a risk of new ADs (known as ‘secondary’ autoimmune diseases) after both autologous and allogeneic HSCT. In recognition of these ‘late effects’, the EBMT and other organisations have produced recommendations for long-term follow-up of patients who should have life-long follow-up by clinicians familiar with ‘late effects’ following HSCT, irrespective of where the procedure has been performed [6–9].

Transplant centres should have approval of their ‘quality’ by external accreditation organisations (Joint Accreditation Committee of ISCT & EBMT, JACIE, or its North American equivalent, the Foundation for Cellular Therapy, FACT), and there is published evidence supporting improved safety and survival outcomes with accreditation [10, 11].

## Haematopoietic stem cell transplantation (HSCT) for ADs

Over the last 20 years, HSCT has also been used to treat severe ADs, as it is an effective means by which to suppress the abnormal inflammation and associated scarring [1–3]. It is also possible that HSCT can rebuild an altered immune system that does not regenerate the original AD, or at least leads to a milder form and better disease control. To date, several thousand patients with ADs have been treated worldwide with HSCT, with almost 3000 patients registered in the EBMT registry (summarised in Table 1).

**Table 1:** Summary of major categories and types of autoimmune diseases treated with HSCT in the EBMT registry, as per August 2018

Autologous HSCT for each category of autoimmune disease	Total adult patients (1994–2018)	Total children (≤18 years at the time of transplant, 1994–2018)	Total patients treated in the last 6 years (all ages, 2012–2017)
All diseases	**2387**	**162**	**1090**
Neurological diseases			
Multiple sclerosis	1232	27	679
Other neurological diseases	94	3	51
Rheumatological diseases			
Systemic sclerosis	534	12	222
Systemic lupus erythematosus (SLE or ‘lupus’)	89	18	9
Other connective tissue diseases	46	5	8
Inflammatory arthritis (including rheumatoid arthritis)	97	67	8
Vasculitis (inflammation of the blood vessels)	39	4	10
Inflammatory bowel diseases			
Crohn’s and other	177	15	83
Haematological autoimmune diseases (causing low blood counts)			
Autoimmune cytopenia, haemolytic anaemia, Evans syndrome, neutropenia and other	41	8	8
Type 1 diabetes mellitus	20	0	3
Miscellaneous other autoimmune diseases	18	3	9

The table summarises activity in autologous HSCT, which accounts for the vast majority of procedures.

Since 1996, 145 allogeneic transplants (from related and unrelated donors) have been performed for autoimmune diseases, approximately three quarters (73%) in children under the age of 18 for a range of haematological, rheumatological and other conditions

Bold type denotes the sum total for each column

The main ADs treated are currently relapsing remitting MS, systemic sclerosis (SSc), systemic lupus erythematosus (SLE or ‘lupus’) and Crohn’s disease. Other rare immune disorders have been treated in smaller numbers. The vast majority of patients have been treated with autologous HSCT, with MS, SSc and Crohn’s disease being the main current indications, reflected by the registrations for the years following the publication of the EBMT ADWP guidelines and recommendations [12] in 2012 (Table 1). A small proportion of patients have received allogeneic HSCT, predominantly for rare diseases in the paedatric age group.

**Fig. 2:**
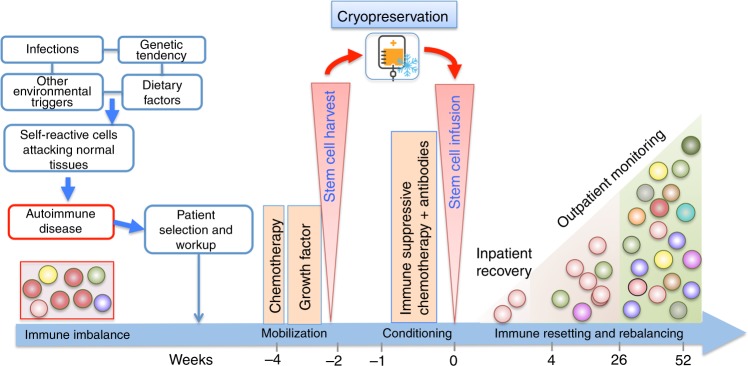
Mechanism of immune re-setting with autologous HSCT in autoimmune diseases

It should be emphasized that HSCT is not a form of ‘regenerative’ stem cell therapy in the same way as many scientists hope that stem cells may be used to rebuild new organs. HSCT may lead to ‘re-setting‘ or ‘re-booting’ of the immune system, which in turn may lead to improvement in damaged organs and tissues. HSCT works best if active inflammation can be switched off, but the effect of HSCT may be limited (or nothing at all) if there is no active inflammation. There is no proof that the blood stem cells can directly rebuild specialised tissues, although in the absence of inflammation some damaged tissues and organs may heal over time. The potential mechanism by which autologous HSCT leads to a re-setting or re-balancing of the immune system in autoimmune disease is summarised in Fig. 2.

Although HSCT is common in many haematology and oncology departments, use of HSCT in ADs is relatively rare and only a small number of transplant centres in each country have a good level of experience in working with disease specialists to select the most appropriate patients with AD and then deliver the HSCT treatment as safely as possible.

The type of HSCT almost always used in patients with severe ADs is autologous HSCT (or ‘auto-transplant’). Auto-transplant is more safely delivered compared with allogeneic HSCT (or ‘allo-transplant’), which is a much more complex procedure requiring a donor. Even so, there are significant risks and toxicities with autologous HSCT, which are an important consideration, especially if the AD has already caused significant organ damage and disability. In view of these toxicities and risks, it is very important that patients have a full discussion with their transplant and autoimmune disease specialist teams.

The individual patient’s response to HSCT is dependent on the specific AD and the stage of the disease at the time of treatment e.g., whether the inflammatory disease process can be ‘switched off’ before it has become irreversible or has caused permanent organ damage. Some patients have had very profound responses lasting many years, far more than expected from treatment with standard therapies, but others respond less well or experience a relapse of their AD soon after the transplant. The benefit of HSCT in some ADs (MS, SSc and Crohn’s disease) has been shown in randomized controlled trials (RCTs) against the best available conventional therapy for an AD. However, there is always need for more evidence to help improve outcomes, and, when possible, AD patients should be treated on ethically approved clinical studies.

## Specific advice to patients considering HSCT for their AD

We recommend that before starting treatment, patients should inform themselves firstly about current disease and treatment guidelines, secondly about the professional competence of the clinicians and thirdly the experience and accreditation status of the transplant centre.

The European Society of Blood & Marrow Transplantation (EBMT) has published a number of guidelines and recommendations for the use of HSCT in ADs [11–13]. Other organisations and individual health professionals have also made recommendations in specific diseases [14–17]. Whereas these guidelines are primarily intended for a professional readership and therefore written in a technical language, they are openly accessible to all.

Timing of HSCT is central to decision-making. The severity of the autoimmune disease, its treatment-resistance and availability of other treatment options are essential considerations. However, HSCT needs to be considered before organ damage caused by the autoimmune disease becomes irreversible (limiting benefits) and before the risks of HSCT are significantly increased by compromised vital organ function. Balancing potential benefits and risks for individual patients is often complex and requires disease specialists and transplant clinicians to work closely together.

The EBMT recommend that patients ask their transplant and disease specialists to explain any aspect of the guidelines, including whether treatment is in line with the published recommendations.

Treating clinicians should be active in the EBMT and/or other national and international professional societies, which means that they should follow published guidelines, report their outcomes to the EBMT (or equivalent professional society) and maintain their professional competencies in this highly specialized field. EBMT membership information is available from www.EBMT.org and accreditation status on www.JACIE.org websites.

Importantly, patients should ask about short- and long-term side-effects of the treatment and the level of risk of death from the treatment and the severe AD to make a balanced decision. Patients should ask their transplant and disease specialists about the experience of their transplant unit in general, whether the transplant unit is accredited (by JACIE, FACT or equivalent) and their specific experience of treating your particular AD with HSCT. Ideally, the transplant centres should have accreditation and have a good track record of experience and academic publication in the disease for which HSCT is being considered.

Patients may also want to ask for an independent specialist opinion or whether their case has had a documented discussion with a wider group of professionals within a multidisciplinary team setting (i.e., an MDT meeting). Ideally patients should be seen and followed up in a combined clinic involving both disease and transplant specialists. Updates on specific ADs are provided on the EBMT websites listed at the end of this position statement.

One exception for being treated outside established guidelines is treatment on a clinical trial approved by a research ethics committee (REC) or institutional review board (IRB), which may, for example, be testing whether a new technique might provide an advantage over existing treatments. In this instance, patients will be counselled and asked to provide an informed consent for the trial.

Even if patients are not on a clinical trial, full informed consent for treatment and also for data reporting is an EBMT requirement for good clinical practice and data protection. In order for the transplant community to gain a better understanding of the long-term side-effects of this treatment, it is important that transplant centres report data relating to transplant treatments to the EBMT or an equivalent registry. In the EBMT, patient data will be handled in an anonymous (and/or pseudonymised) fashion and in line with current general data protection regulation (GDPR).

**Fig. 3:**
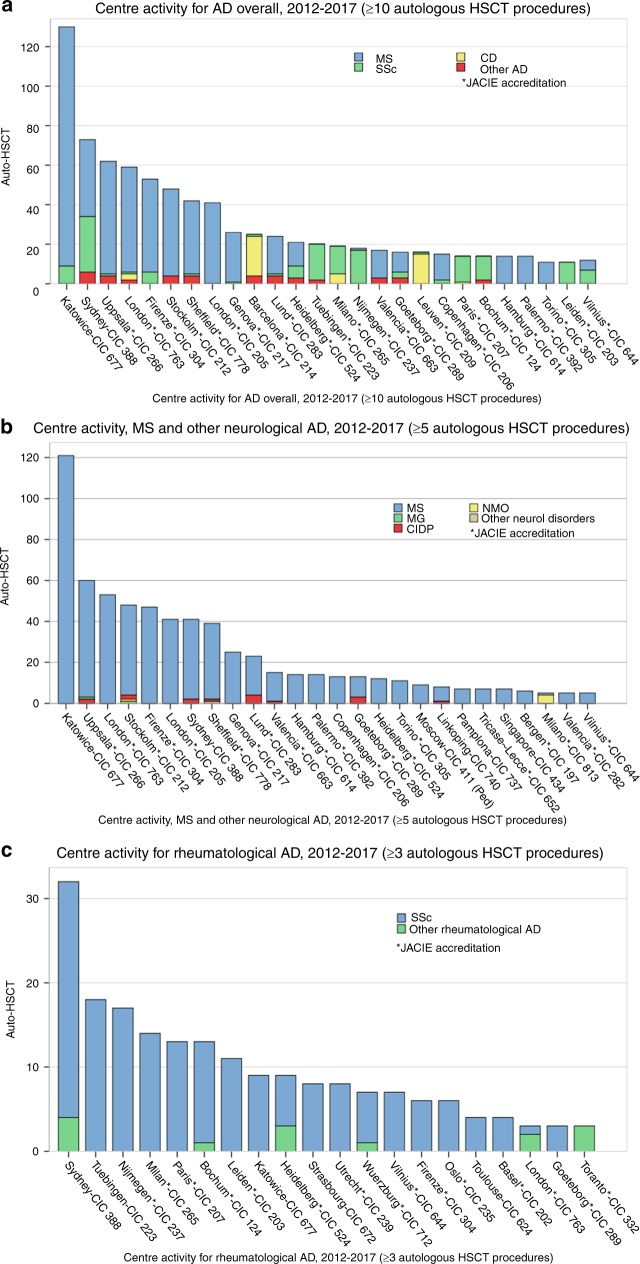

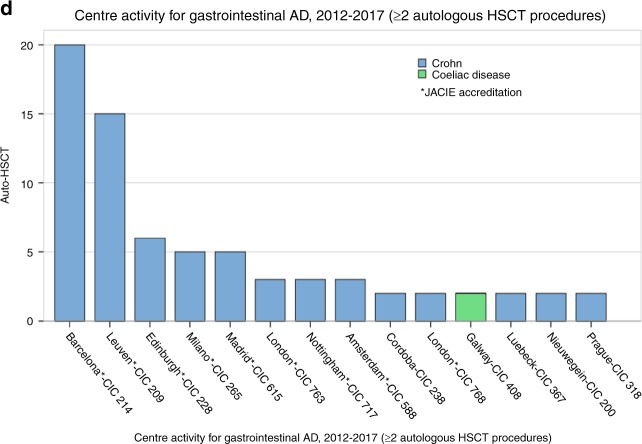
**a**–**d** Activity and JACIE accreditation status in EBMT reporting centres for autologous HSCT in autoimmune diseases. **a** Overall. **b** MS and neurological diseases. **c** Systemic sclerosis and rheumatological diseases. **d** Crohn’s disease and gastrointestinal diseases. Activity is for autologous procedures from 2012–2017 inclusive, i.e., reflecting practice after the publication of EBMT ADWP Guidelines [12]. Accreditation status is indicated by asterisks as active and/or with re-accreditation in progress as per December 2018 (www.JACIE.org). Centre identities according to CIC code are available on https://www.ebmt.org/ebmt-membership-list. The information is primarily intended as a resource for clinicians in transplant centres and disease specialists who wish to make contact with experienced EBMT centres for advice, support, referral or other partnership arrangements aimed at optimising patient care whilst accommodating geographical considerations. The information may also be useful to patients and other carers; however, independent self-referral is strongly discouraged. *MS* multiple sclerosis, *SSc* systemic sclerosis, *CD* Crohn’s disease, *MG* myasthenia gravis, *CIDP* chronic inflammatory demyelinating polyneuropathy, *NMO* neuromyelitis optica

Figure 3a–d summarises recent activity and JACIE accreditation status in EBMT reporting centres for autologous HSCT procedures in ADs, both overall and for specific disease categories. The information is primarily intended as a resource for clinicians in transplant centres and disease specialists who wish to make contact with experienced EBMT centres for advice, support, referral or other partnership arrangements aimed at optimising patient care whilst accommodating geographical considerations. The information may also be useful to patients and other carers; however, independent self-referral is strongly discouraged.

## Treatment abroad

Patients should do their best to enquire whether treatment is available within their own health service, and, if not, they should question why. It may be that treatment is available under specific circumstances that do not cover specific types of AD or a disease specialist may not consider it a best option. If treatment is planned outside of their home country patients should think carefully about their decision, as it may not only have financial implications, but there may be exposure to unnecessary risks to their health and well-being. If treatment is unavailable in a patient’s home country, it is more satisfactory if they travel and receive treatment with the full support of a disease specialist and transplant specialist who knows them well and is familiar with the centre providing the treatment, or at least their general practitioner. This may enable direct communication with transplant and disease specialist teams undertaking the transplant. Independent self-referral by patients is strongly discouraged.

Even if the initial phase of the transplant is a success, patients should be aware that risks of treatment, such as infection and organ damage, last for many months and, in some cases, years after the transplant procedure. As with HSCT in other settings, ongoing follow-up and support is recommended, along with the need for rapid self-referral to a locally available transplant or other specialist haematology unit in case of infections or other emergencies. The follow-up should include the provision of monitoring for ‘late effects’ of HSCT. These aspects of essential care may be compromised if initial HSCT treatment is delivered outside of their home country, especially if communication between clinicians is limited. It is, therefore, important that patients specifically address these issues prior to HSCT treatment as they may require ongoing engagement with clinical services near their home (in both planned or unplanned settings, i.e., emergency or urgent care).

Following return to home, some health services may not support monitoring and follow-up following HSCT, and this may lead to further financial commitment from the patient. The EBMT therefore recommend that before proceeding to transplants, patients carefully assess arrangements and support for medium- and long-term monitoring and follow-up in their locally available health services.

### Summary

- Haematopoietic stem cell transplantation (HSCT) has been used in patients with severe autoimmune and inflammatory diseases whose response to standard treatment options has been limited and associated with a poor long-term prognosis in terms of survival and/or disability. The vast majority of patients have received autologous HSCT, with the use of allogeneic HSCT being rare and mainly in paediatric patients.
- Although a wide range of autoimmune diseases have been treated, the evidence base is greatest for the use of autologous HSCT in relapsing remitting MS, diffuse systemic sclerosis and Crohn’s disease.
- HSCT may work by ‘re-setting‘ or ‘re-booting’ the immune system, which in turn may lead to improvement in damaged organs and tissues. HSCT works best if active inflammation can be switched off, but the effect of HSCT may be limited (or no benefit at all) if there is no active inflammation. There is no proof that the blood stem cells can directly rebuild specialised tissues, although the absence of inflammation may enable some damaged tissues and organs to heal over time.
- Compared with most standard treatments, HSCT is associated with greater short-term risks, including a risk of treatment related mortality (TRM), and long-term complications (so called ‘late effects’) due to the intensity of the treatment. The risk of complications is higher with autologous HSCT in some types of diseases and in allogeneic HSCT. Risks increase with age, more advanced disease and the presence of other conditions which affect patients’ fitness. Decisions should be individualised for each patient.
- Before decisions to proceed with HSCT can be made, it is essential to carry out a careful joint appraisal of potential benefits and risks by disease and transplant specialists working closely together with patients and carers. Alternative non-transplant treatments should always be a consideration in decision-making.
- The EBMT and other professional societies have summarised the evidence and produced guidelines and recommendations as a resource to support centres and good clinical practice.
- HSCT should be conducted in accredited units with robust arrangements for early referral for urgent care after the transplant and long-term follow up with both disease and transplant specialists. Centres should routinely submit data on transplanted patients to international registries, such as EBMT.
- Treatment and follow-up on a clinical trial should always be considered, if available within a reasonable geographical reach of the patient’s home.
- Patients considering HSCT treatment abroad for their AD are strongly advised to ensure that expectations in this position statement are met by the centre offering treatment and that robust referral and follow-up arrangements are made with their local disease and transplant specialists. Independent self-referral is strongly discouraged.

## Appendix

**The EBMT Autoimmune Diseases Working Party (ADWP)**

https://www.ebmt.org/working-parties/autoimmune-diseases-working-party-adwp#

The EBMT ADWP is dedicated to promoting clinical activities, teaching and performing translational research. Our focus is mainly on autologous/allogeneic HSCT, together with of novel forms of cellular therapy and new immunosuppressive drugs in a specific challenge to induce tolerance, with the ultimate objective of better understanding the possibility of resetting the immune response during treatment of severe autoimmune diseases. The EBMT ADWP also has the strategic task of interacting with other autoimmune diseases (AD) specialists and their respective scientific societies.

**The EBMT Nurses Group**

https://www.ebmt.org/nurses-group

The EBMT Nurses Group (NG) plays an essential role in haematology and haematological stem cell transplantation nursing. The group was created 33 years ago and now has over 750 members in more than 60 countries worldwide with a principal nurse identified in almost each EBMT centre. The EBMT NG’s mission is to enhance and value the nurses’ role all over the world, supporting and sharing knowledge through communication, advocacy, research, training and education. The group is dedicated to improving the care of patients receiving stem cell transplantation and works towards promoting excellence in care through recognizing, building upon and providing evidence-based practice.

**The EBMT patient, family and donor committee**

https://www.ebmt.org/patient-family-and-donor-committee

The objectives of the EBMT patient, family and donor committee include supporting national patient advocacy groups, providing a pan-European platform for patient advocacy groups including a patient dedicated link on EBMT website and providing advice on how to optimise EBMT patient centred activities. In addition, the committee aims to increase the numbers of international patients, family members and donors to access the EBMT patient, family and donor day at the annual meeting by promoting the use of online webcasts and translation.

**Joint Accreditation Committee ISCT-Europe & EBMT (JACIE)**

https://www.ebmt.org/accreditation/about-jacie

https://www.ebmt.org/information-patients-donors

JACIE develops and maintain global standards for the provision of quality medical and laboratory practice in cellular therapy. Based on these standards, JACIE offers accreditation to transplant programmes in order to encourage health institutions and facilities to establish and maintain quality management systems impacting on all aspects of their activities and to engage in continuous improvement. JACIE's primary aim is to promote high-quality patient care and laboratory performance in collection, processing and transplantation centres through an internationally recognised system of accreditation.

**Paris EBMT Office**

More technical information for clinicians, including contact with key centres, is available from the Paris EBMT Office via Manuela Badoglio, ADWP Study Coordinator, manuela.badoglio@upmc.fr who has access to haematologists and disease specialists working in your region and countries via the EBMT registry and can liaise with members of the EBMT.
